# MYC amplification in subtypes of breast cancers in African American women

**DOI:** 10.1186/s12885-018-4171-6

**Published:** 2018-03-09

**Authors:** Tammey J. Naab, Anita Gautam, Luisel Ricks-Santi, Ashwini K. Esnakula, Yasmine M. Kanaan, Robert L. DeWitty, Girmay Asgedom, Khepher H. Makambi, Massih Abawi, Jan K. Blancato

**Affiliations:** 1 Department of Pathology, Howard University College of Medicine, Howard University Hospital, 2041 Georgia Avenue Rm. 1M-06, Washington DC, NW 20060 USA; 2 Department of Oncology, University of Massachusetts Medical School, 373 Plantation street Suite# 318, Worcester, MA 01581 England; 3 Cancer Research Center, Department of Biological Sciences, Hampton University, 100 E. Queen Street, Hampton, VA 23668 USA; 4 Department of Pathology, Immunology and Laboratory Medicine, University of Florida College of Medicine, P.O. Box 100275, 1600 SW Archer Road, Gainesville, FL 32610-0275 USA; 5 Department of Microbiology, Howard University College of Medicine, 2041 Georgia Avenue Rm. 1M-06, Washington DC, NW 20060 USA; 6 Department of Surgery, Howard University Hospital, 2041 Georgia Avenue, Washington DC, NW 20060 USA; 7 Department of Medicine, Howard University Hospital, 2041 Georgia Avenue, Washington DC, NW 20060 USA; 8 Department of Biostatistics, Bioinformatics, and Biomathematics, Lombardi Comprehensive Cancer Center, Georgetown University, 4000 Reservoir Road, Washington, DC, NW 20057 USA; 9 Inherited Cancer Program, GeneDx, 207 Perry Pkwy, Gaithersburg, MD 20877 USA; 10 Department of Oncology, Lombardi Comprehensive Cancer Centre, Georgetown University Medical Centre, 3800 Reservoir Road, Washington DC, NW 20007 USA

**Keywords:** MYC, FISH, Gene amplification, Breast cancer subtypes

## Abstract

**Background:**

MYC overexpression is associated with poor prognosis in breast tumors (BCa). The objective of this study was to determine the prevalence of MYC amplification and associated markers in BCa tumors from African American (AA) women and determine the associations between MYC amplification and clinico-pathological characteristics.

**Methods:**

We analyzed 70 cases of well characterized archival breast ductal carcinoma specimens from AA women for MYC oncogene amplification. Utilizing immune histochemical analysis estrogen receptor (ER), progesterone receptor (PR), and (HER2/neu), were assessed. Cases were Luminal A (ER or PR+, Ki-67 < 14%), Luminal B (ER or PR+, Ki-67 = > 14% or ER or PR+ HER2+), HER2 (ER-, PR-, HER2+), and Triple Negative (ER-, PR-, HER2-) with basal-like phenotype. The relationship between MYC amplification and prognostic clinico-pathological characteristics was determined using chi square and logistic regression modeling.

**Results:**

Sixty-five (97%) of the tumors showed MYC gene amplification (MYC: CEP8 > 1). Statistically significant associations were found between MYC amplification and HER2-amplified BCa, and Luminal B subtypes of BCa (*p* < 0.0001), stage (*p* < 0.001), metastasis (p < 0.001), and positive lymph node status (*p* = 0.039). MYC amplification was associated with HER2 status (*p* = 0.01) and tumor size (p = 0.01). High MYC amplification was seen in grade III carcinomas (MYC: CEP8 = 2.42), pre-menopausal women (MYC: CEP8 = 2.49), PR-negative status (MYC: CEP8 = 2.42), and ER-positive status (MYC: CEP8 = 2.4).

**Conclusions:**

HER2 positive BCas in AA women are likely to exhibit MYC amplification. High amplification ratios suggest that MYC drives HER2 amplification, especially in HER2 positive, Luminal B, and subtypes of BCa.

## Background

A significant racial disparity exists in the presentation and outcome of breast cancer (BCa) between African American (AA) women and non-Hispanic white women in the United States. Despite the lower incidence of BCa among AAs (124.3 vs 128.1 per 100,000), the death rate is higher in non-Hispanic AA women (31.0 vs 21.9 per 100,000) [1]. Many Biological and non-biological factors are thought to contribute to these disparities, including access to health care, socioeconomic factors, cultural issues, hormones, reproductive influences, tumor characteristics, growth factors, cell cycle proteins [2], tumor suppressor genes, and chromosomal abnormalities [3, 4].

BCa is a heterogeneous disease consisting of different genetic, cellular, and molecular subtypes, distinct biological entities with unique clinical characteristics [5, 6]. On the basis of intrinsic gene-based signatures using transcriptional profiling, BCa has generally been subdivided into 4 major subtypes: luminal, HER2-enriched, basal-like, and normal breast tumors. Many BCa with basal-like phenotype tumors are ER negative, PR negative, and HER2 negative or triple negative (TNBC) [5.6]. TNBC account for 10 to 20% of all BCa cases and are more prevalent among AA, premenopausal women and women with the BRCA1 mutation. TNBCs tend to be aggressive tumors with poor prognosis in part because no effective targeted therapies have been identified for this BCa subtype [7].

MYC, a multifunctional oncogene located on human chromosome 8q24.21, has been shown to be amplified and overexpressed in many types of human cancers, including ovarian cancer, esophageal cancer, neuroblastoma, sarcoma, lung cancer, and BCas [8]. Depending upon the type of malignancy, the frequency of alterations in MYC varies between 1 and 94% at the cryptogenic level [9]. In a meta-analysis of 29 studies of breast ductal carcinomas, MYC amplification, defined as 2-fold increase in gene copy number, was found in approximately 16% of cases [10]. Amplification of MYC is associated with poor prognosis, high-grade BCas, and early relapse [11, 12].

Identified as a downstream target of HER2, MYC activates various kinase-signaling pathways that may be regulated by ER or PR [13–15]. Many studies have shown that MYC-amplified BCas also harbor HER2 amplification, suggesting a co-amplification [16]. These studies demonstrate that co-amplification of MYC and HER2 augments the development of aggressive tumors with enhanced self-renewal and tumor propagating characteristics. Nevertheless, the underlying mechanism of the interaction between HER2 and MYC that contributes to mammary oncogenesis remains poorly understood [17].

In light of the above, we analyzed a series of well characterized archival invasive ductal carcinoma specimens in order to better understand which BCa subtypes are more likely to be driven by MYC with potential co-amplification of HER2. The objective of the study was to determine the prevalence of MYC amplification in BCa tumors from African American (AA) women and determine the association between MYC amplification and BCa clinic-pathological characteristics.

## Methods

### Specimen acquisition

This retrospective study was based on anonymized, formalin-fixed, paraffin-embedded FFPE archival BCa tissues (e.g. triple negative BCa, Luminal A, Luminal B, and HER2 positive) were obtained from Howard University Hospital Department of Pathology in Washington, DC. They were collected over a ten year period of time ranging from 2000 to 2010. IRB approval for the conduct of this study was received from Howard University’s Institutional Review Board and designated as exempt 15CMED-53. A total of 70 samples were analyzed. Samples were selected based on the availability and adequacy of FFPE tissue blocks for further study. Five μm thick sections were cut and areas of well-preserved carcinoma in a Hematoxylin and Eosin (H&E) orienting slide from each block were marked for study through hybridization and microscopy. Demographic and clinic-pathological data were obtained through the Howard University Cancer Center Tumor Registry.

### Fluorescence in situ hybridization (FISH)

Fluorescence in situ hybridization (FISH) techniques were used to assess MYC amplification in tumor specimens as previously described in our previous study (18). Briefly, the orange fluorophore directly-labeled MYC probe (CymoGen DX, NY USA) and a green labeled centromere 8 probe (Empire Genomics, NY, USA) were used to make a FISH probe mix. The slides were then baked overnight at 60 °C to adhere the tissues to the slides. Slides were subsequently deparaffinized by consecutive 10-min xylene washes before being rehydrated through 100% ethanol incubation for 10 min at room temperature. The tissue on the slides were then permeabilized and digested using the Abbott (Naperville, IL) tissue digestion kit containing pepsin, according to manufacturer’s instruction. The sections were washed with wash buffer after consecutive pretreatment and protease digestion. The probe was denatured at 74 °C for 10 min and immediately transferred to an ice bucket. The denatured probe was then added to the marked area on the slides and was sealed with rubber cement. The slides and the probe were co-denatured for 8 min in HYBrite heat plate (Vysis, Naperville, Illinois) at 85 °C for 8 min and then incubated for 16 to 24 h at 37 °C. Coverslips were removed and the slides were Post Washed in 2X SSC hybridization buffer for 2 min at 73 °C and transferred to 2X SSC at room temperature for 5 min. The slides were air dried in the dark for 1 h in an upright position. Lastly, the slides were counterstained with DAPI (4′, 6-diamidino-2-phenylindole) from Sigma Aldrich and were viewed with a Zeiss Axioscope fluorescence microscope and imaged with Applied Imaging (Pittsburgh, PA) Cytovision software.

### Signal enumeration

**Fig. 1:**
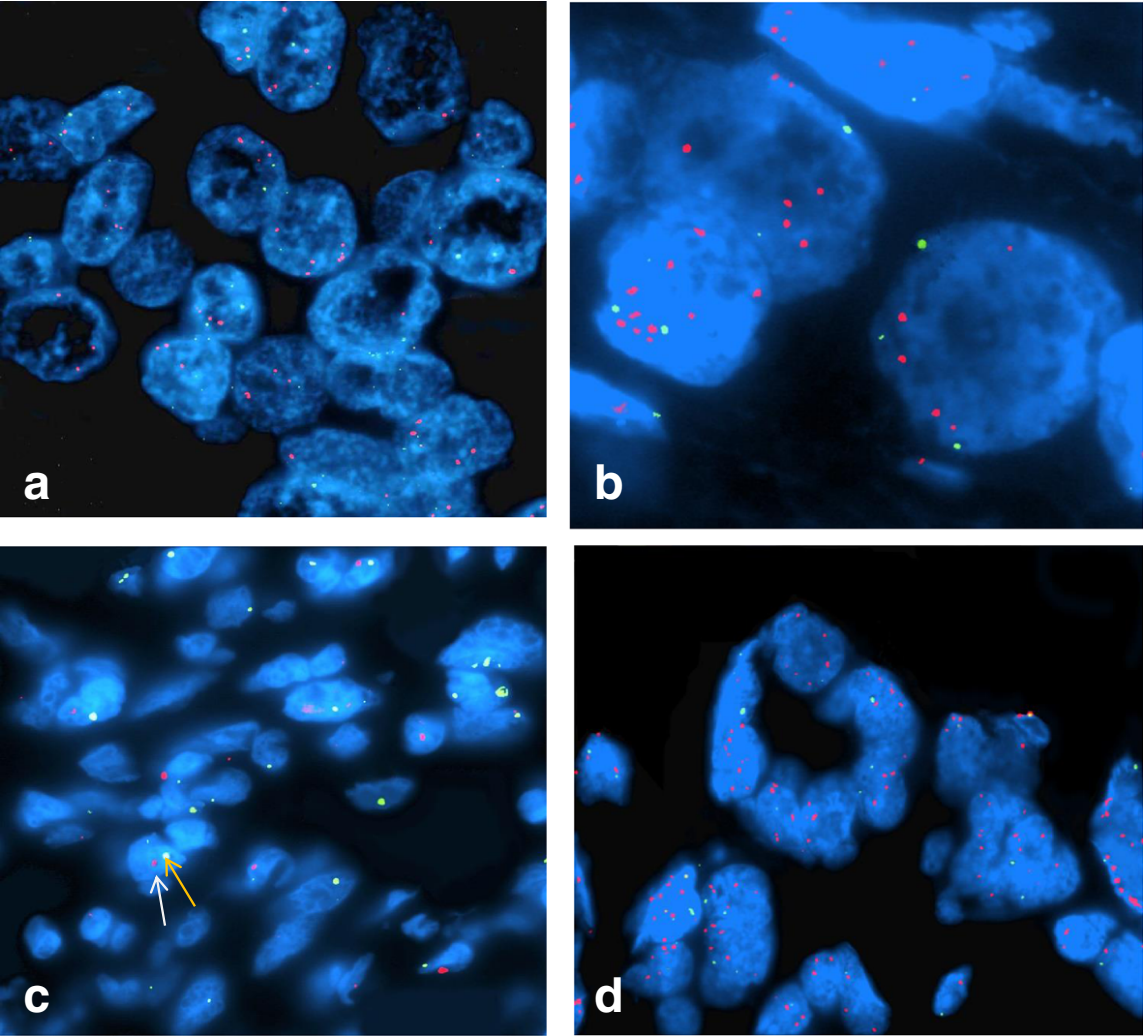
FISH hybridization of MYC in breast tumor tissues. The FISH probe for MYC is labeled with a red fluorochrome, and the normal control signal for the chromosome 8 centromere is labeled in green. The nuclei of the cells are visualized via DAPI counterstaining. **a** 1:3 ratio of MYC to centromere signals indicating low amplification is shown. **b** 1:5 ration of MYC to centromere signals indicating moderate amplification is shown. **c** 1:1 copy ratio of MYC to centromere signals indicating no amplification of the MYC gene is shown. **d** 1: 8 copy ratio of MYC to centromere signals indicating high amplification is shown in the panel

FISH images were acquired using a Zeiss Axioscope fluorescence microscope equipped with a camera and multiple fluorescence filter sets with three color filter set DAPI, FITC and TRITC. The slides were screened with 100× oil objective lens through the DAPI filter to determine the cell area by observing the area of interest marked by the Pathologist. The centromeres were counted as green signals whereas the MYC signals were viewed as orange signals with the TRITC filter (Fig. 1). At least 50 cells were enumerated from a single slide when possible. The signal ratio of the centromere to MYC in normal cells was calculated. The normal control was 2:2; an increase in the ratio was considered abnormal or amplified. The results were expressed as number of MYC signals/number of chromosome 8 signals (MYC: CEP8) [18].

### Statistical methods

The statistical analysis software, IBM SPSS Statistics for Windows(Version 22. Armonk, NY: IBM Corp) was used to perform descriptive analysis of clinic-pathological variables and determine the association between MYC amplification (dependent variable) and prognostic clinic-pathological characteristics such as ER, PR, and HER2 positivity, molecular BCa subtype, stage, grade, and tumor size (independent variables). The Chi-square (χ^2^) test or T test, as appropriate, was used to examine bivariate association between MYC-amplification, CK 5/6 expression and clinic-pathologic variables. Age was categorized into two groups with a cutoff of 50 years. The logistic regression analysis or ANOVA, as appropriate, was used to examine independent association between MYC amplification and clinic-pathological variables. Results were reported as odds ratios (OR) along with calculated 95% confidence intervals (CI). Pearson’s correlation was used to determine the correlation between amplification ratio, tumor size, and age of diagnosis.. Kaplan-Meier (K-M) estimate of overall survival were plotted and log-rank test was performed to compare estimates among groups.

## Results

### Clinicopathologic features

Clinicopathological characteristics of the study population are summarized in Table 1. Seventy paraffin-embedded archival tissues of BCa were studied. The average age at diagnosis was 56 years with a minimum and maximum age of 29 and 85 years, respectively. Approximately 37% of the cases were TNBCs; HER2-amplified tumors comprised 11.4% of the tumors in this study; and Luminal A and Luminal B tumors comprised 30% and 21% of the tumors, respectively. ER, PR, and HER2 were positive in 50%, 45.7%, and 18.6% of tumors, respectively. Most of the tumors were poorly differentiated (74%). The frequency of stage I, II, II, and IV tumors were 23.4%, 41.4%, 22.9%, and 5.7%, respectively.

**Table 1:** Demographic and clinico-pathological characteristics of cases

Characteristics	Mean	Range
Age of Diagnosis (Years)		
	56.36 years	29–85 years
Tumor Size	58.0 mm	2.0–120.0 mm
	Frequency	Percentage
Breast Cancer Subtypes		
HER2	8	11.4
Luminal A	21	30
Luminal B	15	21.4
Luminal B (Ki-67 ≥ 14%)	9	12.9
Luminal B HER2+	6	8.6
Triple Negative	26	37.1
ER Status		
Positive	35	50
Negative	35	50
PR Status		
Positive	32	45.7
Negative	38	54.3
HER2 Status		
Amplified	13	18.6
Not Amplified	57	81.4
Stage		
I	15	23.4
II	29	41.4
III	16	22.9
IV	4	5.7
Grade		
Grade I: Well differentiated	1	1.4
Grade II: Moderately differentiated	15	21.4
Grade III: Poorly differentiated	52	74.3

### FISH analysis of gene amplifications and BCa subtypes

Figure 1 demonstrates representative FISH images with a red labeled MYC probe and a green labeled chromosome 8 centromere probe. Normal, intermediate, and high MYC amplification tissues for these studies are presented. The normal MYC: CEP8 ratio is 1. The average MYC amplification ratio was 2.25. Thirty-six percent of the cases had high amplification ratio (MYC: CEP 8 ratio > 2). High MYC amplification scores were observed in HER2 (3.97 ± 1.99) and Luminal B (3.34 ± 2.11) subtypes, whereas low amplification was seen in TNBC (1.65 ± 0.94) and Luminal A (1.65 ± 0.93) subtypes as shown in Table 2. The mean MYC amplification ratio of HER2, Luminal A, and TNBC differ significantly; however, HER2 and Luminal B do not significantly differ from each other (*p* < 0.001).

**Table 2:** C-MYC Amplification in various breast cancer subtypes

Breast Cancer Subtype	Frequency	Mean	Standard Deviation	Min	Max	Tukey Grouping
HER2	8	3.63	2.27	1.16	7.08	A
Luminal A	21	1.65	2.12	0.94	4.7	B
Luminal B	15	3.19	0.67	1.00	9.28	A
Triple negative	26	1.52	2.27	0.76	3.56	B

ANOVA F-test =8.52; *p* < 0.0001 A = 0.625 B = 1.00

### MYC amplification and HER2/PR/ER status

In all, 20% of the samples were HER2 positive (13% HER2 subtype, 7% Luminal B). The average MYC amplification ratio for HER2 positive BCas was 4.36 (±2.24) and 1.77 (±0.97) for HER2 negative/not amplified BCa cases as presented in Table 3. The mean difference between Her2 + and Her2- tumors was statistically highly significant with *p* = < 0.001 as detailed in Table 3. Average MYC amplification scores did not differ significantly between ER+ and ER-, as well as PR+ and PR- tumors. Regression analysis also revealed an association between the HER2+ subtype (OR = 29.75 95% CI: 2.80–315.56; *p* < 0.0001) and Luminal B HER2 positive subtype (OR = 10.63 95% CI 2.16–52.15; *p* < 0.0001), as well as with HER2 immuno-histochemical expression (OR = 40.62 95% CI 4.82–342.39; *p* < 0.00001). However, no statistically significant association was found between MYC amplification and cancer grade, origin of tumor, tumor size, or age of diagnosis.

**Table 3:** MYC amplification with ER, PR and HER2

Characteristic	N	Mean	Std Dev	Minimum	Maximum	*p*-value
ER						
Negative	35	2.18	1.57	0.76	7.08	
Positive	35	2.33	1.71	0.94	9.28	0.71
PR						
Negative	38	2.47	1.94	0.76	9.28	
Positive	32	1.99	1.14	0.94	5.50	0.23
HER2						
Negative	57	1.77	0.97	0.76	5.00	
Positive	13	4.36	2.24	1.28	9.28	< 0.001

### BCa survival analysis and BCa subtypes

In addition, Table 4 shows the survival analysis data in relation to MYC amplification and clinic-pathological variables. Statistically significant differences in overall survival were found when stratifying by stage (*p* < 0.001), having metastatic disease (*p* < 0.001), and lymph node positivity (*p* = 0.039). However, no statistically significant difference in overall survival was observed among BCa subtypes in this study (Log rank test *p* = 0.201). The mean overall survival was 71.00 months for HER2 subtype (95% CI = 44.09–97.91), 94 months (95% CI = 84.56–103.44) for Luminal B, 67.71 months (95% CI = 52.05–83.36) for Luminal A, and 100.07 months (95% CI = 76.57–123.57) for TNBC subtype.

**Table 4:** Regression analysis of MYC expression and clinico-pathological variables

	C-MYC Amplification				Odds	95% CI		
	< 2		> 2		Ratio	Lower	Upper	*p*-value
Breast Cancer Molecular Subtype								
Luminal A	17	81.0%	4	19.0%	ref.			
Luminal B	4	28.6%	10	71.4%	10.63	2.16	52.15	< 0.0001
HER 2+	1	12.5%	7	87.5%	29.75	2.80	315.56	< 0.0001
Triple Negative Breast Cancer	23	85.2%	4	14.8%	0.74	0.16	3.38	0.72
Estrogen Receptor Status								
Positive	21	60.0%	14	40.0%	ref.			
Negative	24	68.6%	11	31.4%	0.69	0.26	1.84	0.31
Progesterone Receptor Status								
Positive	21	65.6%	11	34.4%	ref.			
Negative	24	63.2%	14	36.8%	1.11	0.42	2.98	0.52
HER2 Status								
Negative	44	77.2%	13	22.8%	ref.			
Positive	1	7.7%	12	92.3%	40.62	4.82	342.39	< 0.0001

Table 5 demonstrates that differences in mean survival were not found when stratifying by MYC amplification (*p* = 0.265). .

**Table 5:** Survival analysis of clinico-pathological variables and MYC amplification

			95% CI		
	Mean Months	Std. Error	Lower Bound	Upper Bound	*p*-value
Stage					
1	119.59	9.62	100.73	138.45	
2	92.03	7.75	76.85	107.21	
3	98.25	15.12	68.61	127.89	
4	18.25	6.02	6.45	30.05	*p* < 0.001
Size (T)					
T1	89.35	4.53	80.47	98.23	
T2	100.60	9.98	81.04	120.17	
T3	82.50	16.74	49.69	115.32	*p* = 0.06
Distant Metastases					
No Metastatic Disease (0)	114.88	7.06	101.05	128.71	
Metastatic Disease (1)	38.78	13.97	11.40	66.16	*p* < 0.001
Lymph Nodes					
Lymph Node Negative (0)	124.65	8.29	108.40	140.90	
Lymph Node Positive (1)	86.88	10.33	66.64	107.11	*p* = 0.039
Breast Cancer Molecular Subtype					
Luminal A	67.71	7.99	52.05	83.36	
Luminal B	94.00	4.82	84.56	103.44	
HER 2+	71.00	13.73	44.09	97.91	
Triple Negative Breast Cancer	100.07	11.99	76.57	123.57	*p* = 0.201
C-MYC Amplification					
< 2	93.96	8.96	76.41	111.52	
> 2	117.03	9.98	97.47	136.59	*p* = 0.265

## Discussion

An association between MYC amplification and HER2 overexpressing tumors in AAs was found in this pilot study of AA patients. Importantly, cases of Luminal B with positive HER2 expression exhibited the highest MYC amplification ratios. Specifically, HER2/neu receptor positive cases had 4.5 more copies of MYC than HER2/neu negative cases, a difference that suggests a significant association between MYC amplification and hormone receptor positivity in HER2 positive Luminal B BCas (*p* = 0.01). Luminal A HER2 positive BCas did not demonstrate high MYC amplification. We did not observe any association between MYC amplification and tumor grade, tumor size, or age at diagnosis similar to other studies [18–20].

In BCas, amplification of MYC is associated with poor prognosis, high-grade, and early relapse [11–13]. The distribution of MYC amplification varies by BCa subtypes and MYC amplification has been shown to be associated with BCa molecular subtypes and hormonal receptor status. High MYC amplification (MYC: CEP8 ratio > 2) has been reported in HER2 and Luminal B subtypes of BCa whereas low MYC amplification scores were observed in Luminal A and TNBC subtypes [19, 20]. Another study showed high MYC amplification in TNBCs, particularly incases demonstrating a basal-like phenotype [21–23].

The majority of analyses have shown that MYC-amplified BCas also harbor HER2 amplification, suggesting a co-amplification mechanism (16). Consistent with these studies, we also demonstrated that HER2 positive BCas in this population of AA women were more likely to exhibit MYC amplification. High amplification ratios may suggest that increases in MYC activity drives HER2 amplification, especially in HER2 positive and Luminal B HER2-amplified subtypes of BCa. Therefore, MYC may play a significant role in steroid receptor dependent as well as independent tumorigenesis.

This study shows low MYC amplification scores in Luminal A and TNBCs. Another recent study measured MYC gene copy number in 94 breast tumors of all subtypes with semi-quantitative multiplex PCR. They did not find any association of amplification with age, tumor size, ER and PR status, local metastases, or histologic grading, but noted that MYC amplification was present in early tumors but not adjacent normal tissues [11]. Laboratory test systems, FISH probe choices, and microscopy factors affect study sensitivity, particularly with low levels of amplification which may not be resolved adequately.

To further explore the relationship between MYC amplification and steroid receptor status, we evaluated the association between MYC: CEP8 ratio and the presence/absence of PR and ER. Our study yielded mixed results consistent with those obtained in previous studies [15, 24]. The mean MYC amplification ratio in PR negative tumors was 0.5 times higher than in tumor tissues that were PR positive; ER positive cases had slightly higher MYC amplification ratios than ER negative cases. However, there was no significant relationship between the PR/ ER receptor status and MYC amplification (*p* = 0.50, 0.57, respectively). Notably, the MYC amplification score was high in tumors that metastasized to brain or bone. Although the precise nature of the ER and PR signaling pathway and its relationship with MYC amplification remain unclear, PR was reported to influence proliferation and cell differentiation, and to exhibit a biphasic effect on cell growth and MYC expression [25]. A more recent study conducted by Wang et al. indicated that MYC expression could be induced by estrogen, involving the integration of MYC between ER and AP-1 at the distal enhancer element suggesting that AP-1 may play a role in induction of the MYC oncogene [26].

Future studies to investigate the role of MYC as a downstream mediator of HER2 may assist in understanding the role MYC gene on growth regulatory genes in different BCa subtypes. Also, studies evaluating differences in MYC amplification between primary breast tumors and metastatic sites in bone, lung, and brain would be useful to determine whether lethality of metastatic disease is related to MYC amplification, especially in minority populations.

## Conclusion

We demonstrate that HER2+ breast cancer tissues from AA women that demonstrate also have a high likelihood to show MYC amplification. High amplification ratios suggest that MYC drives HER2 amplification, especially in HER2 positive BCas including both the HER2 subtype and HER2 + Luminal B subtype. This provides support the concept that MYC plays a part on steroid dependent and independent tumor development.

## Abbreviations

AA: African American
ANOVA: Analysis of Variants
BCa: Breast cancer
CEP: Centromere Enumeration Probe
CI: Confidence Interval
DAPI: 4′,6-diamidino-2-phenylindole
ER: Estrogen Receptor
FFPE: Formalin fixed paraffin embedded
FISH: Fluorescence in situ Hybridization
FITC: Fluorescein isothiocyanate
H & E: Hematoxylin and Eosin
HER2/neu: Receptor tyrosine-protein kinase erbB-2
IRB: Institutional Review Board
MYC: c-Myc gene
OR: Odds Ratio
PR: Progesterone Receptor
TNBC: Triple Negative Breast Cancer
TRITC: Tetramethylrhodamine
